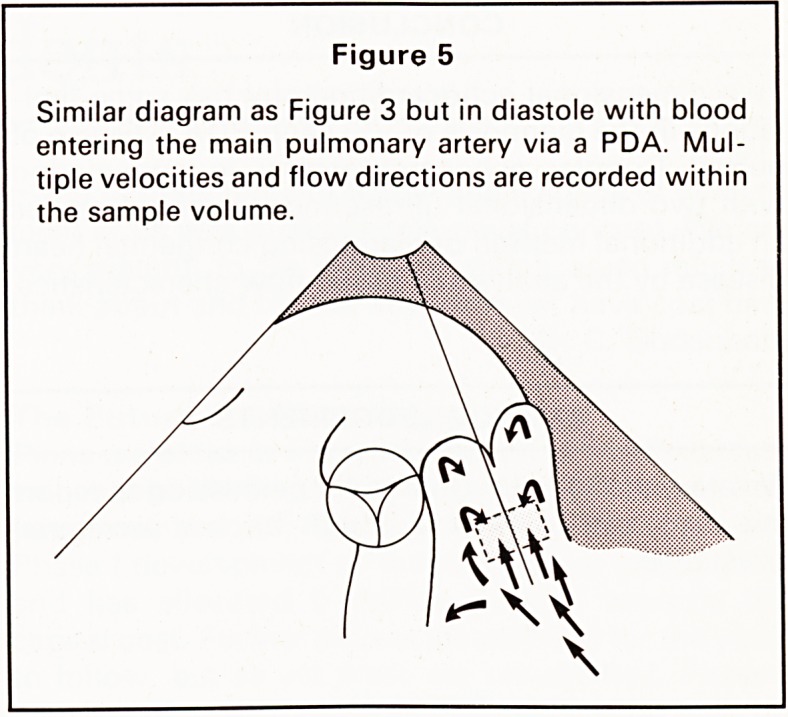# Pulsed Doppler Echocardiography in the Diagnosis of Ventricular Septal Defect and Patent Ductus Arteriosus

**Published:** 1984-07

**Authors:** Graham Buirski, Peter Wilde

**Affiliations:** Department of Radiodiagnosis, Bristol Royal Infirmary; Department of Radiodiagnosis, Bristol Royal Infirmary


Case Report

### Pulsed Doppler Echocardiography in the Diagnosis of Ventricular Septal Defect and Patent Ductus Arteriosus

**Graham Buirski and Peter Wilde**
Department of Radiodiagnosis, Bristol Royal Infirmary

### INTRODUCTION

Anatomical imaging of congenital heart disease can now be made non-invasively using two dimensional echocardiography. The additional application of pulsed Doppler echocardiography can provide information about the direction, timing and quality of flow within the cardiac chambers and great vessels and this functional information can give more accurate diagnosis of shunts and valvular disease.

Two dimensional echocardiography does have some limitations and it is unable to detect small abnormalities beyond the imaging resolution of the apparatus. Ventricular septal defects (VSDs) can only be detected if they are sufficiently large or are associated with certain anatomical malformation (Magherini et al., 1983). Accurate demonstration of a patent ductus arteriosus (PDA) is limited due to echo dropout which makes visualisation of the ductal area difficult (Hagan et al., 1980). The following case report illustrates how pulsed Doppler echocardiography can provide useful clinical information non-invasively in a patient with a normal two dimensional echocardiogram.

### CASE REPORT

R. H. This male infant was born in May 1982 after a normal delivery at 39 weeks' gestation. At 48 hours after birth he became cyanosed during feeding and clinical examination revealed slight dyspnoea with a pansystolic murmur. The electrocardiogram showed right ventricular enlargement and the chest X-ray showed pulmonary plethora consistent with a left to right shunt. An M-mode echocardiogram showed no abnormality.

One week after birth cardiac catheterisation demonstrated a large VSD and a small PDA. His condition improved on medical treatment and he was then discharged from hospital. Over the next 6 months he had recurrent chest infections which required intermittent positive pressure ventilation on one occasion.

In November 1982 he underwent elective closure of the PDA. Postoperatively he developed further chest infections which were treated medically with eventual resolution of a collapsed lower lobe.

He was readmitted in October 1983 for repeat cardiac catheterisation (now aged 16 months) because of persisting breathlessness and failure to gain weight. Two dimensional echocardiography yielded good quality images but no evidence of VSD was found and the PDA was not visualised with certainty. Pulsed Doppler echocardiography examination of the interventricular septum demonstrated localised high frequency turbulent flow diagnostic of a VSD in the perimembranous region (Figures 1a, b). Further examination revealed normal laminar flow in the right ventricular outflow tract below the pulmonary valve but there was turbulent diastolic flow in the main pulmonary artery (Figures 2a, b) indicating a persistent PDA. A 2 mm VSD and a 2 mm diameter PDA were both confirmed by cardiac cineangiography. The patient was discharged to follow up, clinically well on medical therapy.

### DISCUSSION

Many modern two dimensional echocardiography instruments now have an integral pulsed Doppler system which generates its ultrasound beam from within the same real time transducer. It is possible to place accurately in the two dimensional image the site at which the pulsed Doppler signal can be analysed. At this site measurements are made of the velocity and direction of flow in blood within the small volume being examined (Figure 3). The range of frequency shifts (related to velocity) calculated are displayed on a time base line (Figure 4). Normal laminar flow produces a typical appearance where at any time (point x, Figure 4) the velocity of blood moving through the sample volume is similar. The curve will be above or below the base line depending on whether flow is towards or away from the transducer. The simultaneous electrocardiogram enables flow to be related to either diastole or systole.

Turbulent flow is recognised by blood within the

**Figure 1a**

Subcostal 4-chamber view showing the sample volume (SV) positioned close to the membranous part of the interventricular septum (IVS). Left ventricle (LV), right ventricle (RV), left atrium (LA) and right atrium (RA) are shown.

**Figure 1b**

Doppler trace from position shown in Figure 1a. During systole multidirectional high frequency turbulent flow is detected confirming a VSD. Time is on the horizontal axis and frequency shift is on the vertical axis with flow towards the transducer above the centre line and flow away being below the central line.

sample volume travelling at different velocities at the same instant, which produces a wide range of frequency shifts at any one particular time (point y, Figure 4). Multidirectional turbulence can lead to flow towards and away from the transducer being recorded within the sample volume at the same time (Figure 5).

The diagnosis of VSD by Doppler echocardiography is made by demonstrating a localised turbulent systolic jet on the right side of the septum in a left to right shunt and then by following it back through the septum into the left ventricular cavity (Baker, 1980). The presence of turbulent flow over the septum alone is also diagnostic (Magherini et al., 1980). Figure 1b demonstrates systolic bidirectional turbulent flow with the sample volume over the membranous ventricular septum (Figure 1a) confirming the presence of a VSD.

The sensitivity of pulsed Doppler echocardiography in detecting a VSD is 90% with a specificity of 98% (Baker, 1980). The presence of a jet in the right ventricle and not within the septum is non-specific and may occur in conditions such as right ventricular outflow tract obstruction (Magherini et al., 1980).

**Figure 2a**

Parasternal short axis view showing sample volume (SV) in the main pulmonary artery. A possible patent ductus is visualised between the main pulmonary artery and descending aorta. Main pulmonary artery (MPA), right ventricular outflow tract (RVOT), aortic root (AO), left atrium (LA) and descending aorta (DA) are shown.

**Figure 2b**

Doppler trace from position shown in Figure 2a. Systolic laminar flow away from the transducer and multidirectional diastolic turbulent flow are seen. This confirms the patient ducts arteriosus.

The flow across a VSD may be laminar in the presence of large defects or where there is increased pulmonary vascular resistance (Baker, 1980).

In the case of patent ductus arteriosus the turbulent flow demonstrated in the main pulmonary artery (Figures 2a, b) occurs in diastole and is bidirectional, but is predominantly towards the transducer (and pulmonary valve). Flow from the aorta to pulmonary artery is directed through the PDA towards the transducer and pulmonary valve but some blood streams in the opposite direction towards the lungs and causes turbulence. The systolic flow pattern in Figure 2b is produced by laminar flow through the pulmonary valve. It is important to recognise bidirectional flow in the diagnosis of PDA because unidirectional diastolic turbulence towards the transducer may occur in pulmonary regurgitation and away from the transducer in aorto-pulmonary windows (Daniels, 1983).

In our patient the two dimensional echocardiogram was unable to detect the presence of VSD due to its small size, and the patent ductus was not confidently diagnosed on the two dimensional image (Figure 3a).

**Figure 3**

Diagram illustrating the parasternal short axis view in systole. The blood passing through the sample volume in the main pulmonary artery is all moving in the same direction at the same velocity. This corresponds to point x in Figure 4.

**Figure 5**

Similar diagram as Figure 3 but in diastole with blood entering the main pulmonary artery via a PDA. Multiple velocities and flow directions are recorded within the sample volume.

**Figure 4**

The Doppler trace (top) and electrocardiogram (below) allow direction and timing of flow to be assessed. Point x shows laminar flow and point y shows turbulent flow.

### CONCLUSION

Two dimensional echocardiography has some limitations in the diagnosis of VSD and PDA. The use of pulsed Doppler echocardiography in conjunction with two dimensional ultrasound imaging provides an additional method of diagnosing congenital heart disease by the analysis of blood flow characteristics.

### ACKNOWLEDGEMENTS

We are grateful to Dr. Jordan for permission to report this case and to Miss J. Hugh for her secretarial assistance.

### REFERENCES

MAGHERINI, A., ALZOINA, G., WIECHMANN, V. and SANTINI, F. (1980) Pulsed Doppler echocardiography for diagnosis of Ventricular septal defects. *British Heart Journal* **43**, 143–147.

HAGAN, A. D., DISESSA, T. G., BLOOR, C. M. and CALLEJA, H. B. (1983) Two Dimensional Echocardiography: Clinical-pathological correlations in adult and congenital heart disease. Boston, Little and Brown, Chapter 14, p 462.

BAKER, D. W. (1980) Applications of pulsed Doppler frequency. *Rad. Clinics of North America* **18**, No. 1, 92–94.

DANIELS, O. (1983) Doppler Cardiography: Clinical Applications. Symposium—The Hague 19th–21st June (1983).

## Figures and Tables

**Figure 1a Figure 1b f1:**
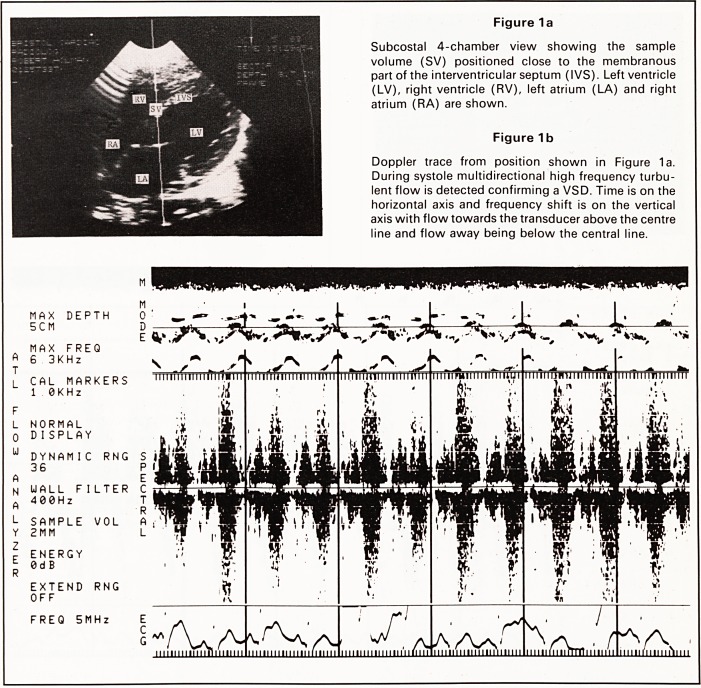


**Figure 2a Figure 2b f2:**
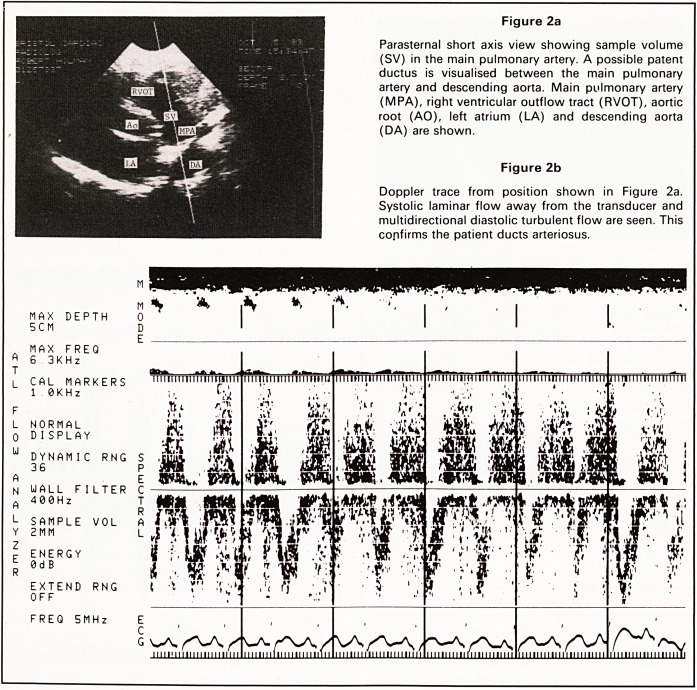


**Figure 3 f3:**
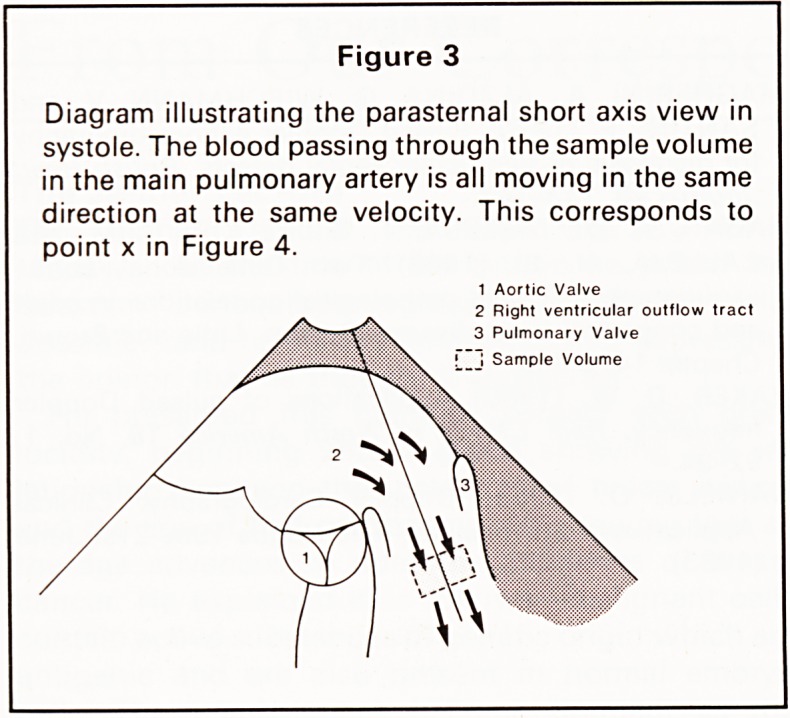


**Figure 4 f4:**
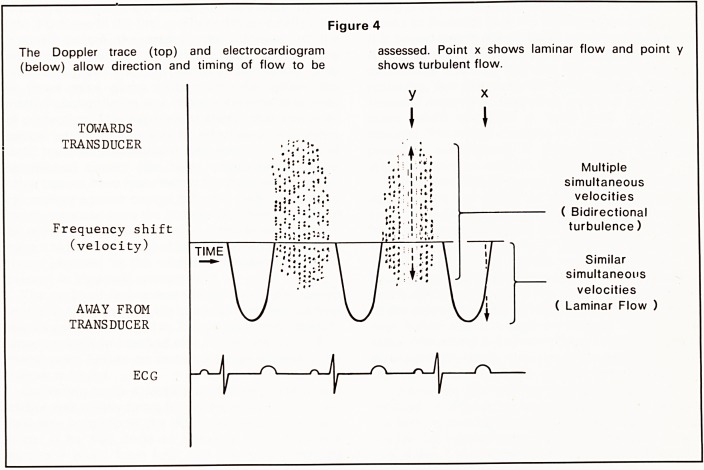


**Figure 5 f5:**